# Dynamics of System States in the Probability Representation of Quantum Mechanics

**DOI:** 10.3390/e25050785

**Published:** 2023-05-11

**Authors:** Vladimir N. Chernega, Olga V. Man’ko

**Affiliations:** 1 Institute of Managment and Digital Technologies, Department of Logistics and Transport System Managment, Russian University of Transport (MIIT), Obraztsova Street, 9/9, Moscow 127994, Russia; mail@vladimirchernega.ru; 2 Lebedev Physical Institute, Russian Academy of Sciences, Leninskii Prospect 53, Moscow 119991, Russia

**Keywords:** entangled probability distributions, entanglement, quantizer operator, dequantizer operator, symplectic tomography, center-of-mass tomography, even and odd cat states

## Abstract

A short description of the notion of states of quantum systems in terms of conventional probability distribution function is presented. The notion and the structure of entangled probability distributions are clarified. The evolution of even and odd Schrödinger cat states of the inverted oscillator is obtained in the center-of-mass tomographic probability description of the two-mode oscillator. Evolution equations describing the time dependence of probability distributions identified with quantum system states are discussed. The connection with the Schrödinger equation and the von Neumann equation is clarified.

## 1. Introduction

The motivation of this work is to show, using a simple example of a quantum oscillator and its evolution, that the oscillator states can be described by conventional probability distributions. The dynamics of the oscillator states are described by the time dependence of the probability distribution identified with the quantum state. This description of the quantum system is valid for all the oscillator systems, including the inverted oscillator. The explicit description of this time evolution of the probability distribution, called the center-of-mass tomographic probability distribution of the two-mode inverted oscillator, is one of the goals of our work. Another objective of this paper is to study the structure of probability distribution functions, which describe the entangled states of the quantum system.

The conventional languages of quantum mechanics and classical mechanics are very different from each other. The language of classical mechanics operates with definitions, such as functions, point-wise multiplication, and probabilities. The quantum mechanical language is much more complicated. It operates with such definitions as operators, density matrices, and state vectors. At the dawn of quantum mechanics, in the time of Dirac, it was even called matrix mechanics because of its mathematical apparatus. There are some phenomena and concepts that have passed from classical mechanics to quantum mechanics. The principle of superposition in quantum mechanics appeared as a natural extension of the phenomenon of wave interference in classical mechanics, and Bohr’s quantization rule was inspired by the classical condition for maximum wave interference, which was applied to the de Broglie wave. Some phenomena of quantum mechanics have no classical analogs, such as spin phenomenon or entanglement of states. It would be interesting to enrich the classical theory with some concepts that appear in quantum mechanics, and that were not previously in the classical theory. One such concept is the entangled probability distribution, which we will consider in this paper.

The states of the quantum system in standard formulation of quantum mechanics [1] are determined either by vectors |ψ〉 in the Hilbert space [2] or by density operators $\hat{\rho }$ acting in the Hilbert space [3]. The vectors in the Hilbert space are associated with wave functions ψ(x) of pure quantum states, and the density operators are associated with pure or mixed states described by density matrices [4] or matrix elements of the density operators in some representations. Different representations of quantum states were constructed, e.g., Wigner functions W(q,p), which are quasiprobability distributions [5,6] that have some properties of probabilistic distributions. In classical mechanics, the system states are described by objects that are probability distribution functions, and their properties are described by conventional probability theory [7]. The probability theory is also used to study different aspects of quantum system properties [8] as well as in connection with quantum mechanical methods applications to other areas of science [9]. Some new aspects of quantum system correlation properties, such as entanglement phenomena, were discussed in [10,11]. The entanglement phenomenon in quantum physics provides the possibility to apply this notion in classical probability theory [12]. The functions that define the states of a quantum system, and that are probability distribution functions, were introduced in [13]; they were named symplectic tomograms, and this representation was called probability representation of quantum mechanics (see also [14,15,16,17,18]). Some mathematical aspects of the probability representation of quantum and classical states were discussed in [19,20]. The possibility of finding the probability representation of quantum states is based on the existence of the invertable map of the density operators of mixed or pure quantum states, which is mapped onto conventional probability distribution functions. We will discuss this map in our paper, using the example of quantum oscillator states.

The tomograms and the entanglement phenomenon in the two-mode squeezed states and two-mode even and odd coherent states were considered in [21]. Stimulated Raman scattering and stimulated Brillouin scattering of light were considered within the frame of the symplectic tomography scheme in [22,23]. Furthermore, the entanglement phenomenon in the processes of stimulated light scattering of different types, and its connection with the probability distribution functions determining the states of photon and phonon modes, was discussed [22,23,24,25]. In [26], the evolution of different kinds of states in the Kerr medium, including maximum entangled states, were theoretically studied within the frame of the optical tomography scheme (which is a partial case of the symplectic tomography scheme). The instability of the reconstructed tomogram determining the state was considered in [27] in connection with the Radon transform properties. In [28], it was shown that, in classical mechanics, the Hermitian operators can be introduced, and the concepts of classical mechanics can be formulated in a language analogous to the quantum mechanics language. In [29], a review of classical probability representations of quantum states and observables is presented. New fundamental aspects of quantum mechanics based on the groupoid approach are investigated in [30]. In [31], the evolution of states of a system containing quantum and classical parts was studied. The cosmology features were considered within the frame of the probability representation of quantum states in [32,33]. The density matrix properties, using the symplectic representation of quantum mechanics, are given in [34]. In some tomographic methods, the quantization is based on the associative star product of the functions; applications of these approaches in different kinds of experiments were discussed in [35,36,37,38,39,40,41,42,43,44].

The idea to construct the probability representation of quantum states is based on the method of mapping operators onto functions called symbols of operators. This method is the same method that is used to construct the Wigner function [5] and other quasidistributions, such as the Husimi function [45] and the Glauber–Sudarshan P-function [46,47].

The aim of this paper is to study properties of the probability representation and to consider the probability distributions, describing the quantum states in the case of continuous variables. We will consider the dynamics of the quantum oscillator states as the dynamics of the probability distributions, including the superpositions of the wave functions and the superposition principle. Moreover, some examples of the probability distributions for continuous variables (called tomographic probability distributions) will be studied for quantum oscillator systems.

The paper is organized as follows: The notion of entangled probability distributions describing the quantum states in the probability representation of quantum mechanics is discussed in Section 2. A specific example of the entangled probability distribution for a two-mode oscillator is considered in Section 3. The time dependence of states in different representations of quantum mechanics is described in Section 4. The probability representation of quantum states is described in Section 5, using the method of quantizer–dequantizer operators, as well as the evolution equation for the probability distributions and other functions corresponding to quasiprobability representations of system states. The symplectic tomography of oscillator system states is discussed in Section 6, and the dynamics of operator symbols for the Hamiltonians, which are quadratic forms of position and momentum operators, are considered in Section 7. The center-of-mass tomography and dynamics of the Schrödinger cat states of the ordinary and inverted two-mode oscillators, including explicit expressions for time evolution of the center-of-mass tomograms, are presented in Section 8. The conclusions and prospectives of the probability representation of quantum mechanics for studying entanglement and dynamics of the states of quantum systems are presented in Section 9.

## 2. Entangled Probability Distributions of Random Variables

In order to discuss the entangled probability distribution notion, we will address the concept of the conditional probability notion using an example of a probability distribution of two random variables P(X,a), where *X* and *a* are real continuous parameters and the non-negative function satisfies the normalization condition

(1) ∫P(X,a)dXda=1.

Then, the conditional probability distribution P(X|a) is related to the function P(X,a) by the Bayes formula

$$P\left(X|a\right)=\frac{P(X,a)}{\int dXP(X,a)},$$

where *a* the parameter describing the condition of measuring variable *X*. The important property of this formula is the normalization condition of the function P(X|a), which reads

(2) ∫dXP(X|a)=1.

This means that for conditional probability distributions, the integration of the function as the function of the random variable *X* gives the result that does not depend on the condition parameter *a*. Analogous properties take place for conditional probability distributions of general random variables, which we will use in our construction of entangled probability distributions in an example of the function of two random variables $X_{1}$ and $X_{2}$.

Following [12], we introduce the concept of separable and entangled probability distributions using the notion of entangled states in quantum mechanics, and, as introduced in [13,16,48], the notion of probability representation of quantum states. In this representation of a quantum system, the density operators of separable states can be written as a convex sum of tensor products of the density operators of the subsystems. Using the probability representation of the density operators, we formulate the new notion in the conventional probability theory using, as an example, the probability distribution of two random variables that are obtained using the invertible map of the density operators, which are mapped onto the probability distributions. Definition: the conditional probability distribution $P(X_{1},X_{2}|a_{1},a_{2})$ is deemed separable if it can be represented as the convex sum of the products of the probability distributions $P^{\left(k\right)}\left(X_{1}|a_{1}\right)$ and $P^{\left(k\right)}\left(X_{2}|a_{2}\right)$ of the form

(3) $$P\left(X_{1},X_{2}|a_{1},a_{2}\right)=\sum_{k}P_{k}P_{1}^{\left(k\right)}\left(X_{1}|a_{1}\right)P_{2}^{\left(k\right)}\left(X_{2}|a_{2}\right).$$

Here, $P(X_{1},X_{2}|a_{1},a_{2})\geq 0$, $P_{1}\left(X_{1}|a_{1}\right)\geq 0$, $P_{2}\left(X_{2}|a_{2}\right)\geq 0$, coefficients $P_{k}\geq 0,\;\sum_{k}P_{k}=1$ and

(4) $$\int P\left(X_{1},X_{2}|a_{1},a_{2}\right)dX_{1}dX_{2}=1.$$

The probability distribution $P(X_{1},X_{2}|a_{1},a_{2})$ is called the entangled probability distribution if it cannot be presented as the convex sum of the form (3), i.e.,

(5) $$P\left(X_{1},X_{2}|a_{1},a_{2}\right)\neq \sum_{k}P_{k}P_{1}^{\left(k\right)}\left(X_{1}|a_{1}\right)P_{2}^{\left(k\right)}\left(X_{2}|a_{2}\right).$$

For separable probability distribution

(6) $$\int P\left(X_{1},X_{2}|a_{1},a_{2}\right)dX_{2}=\sum_{k}P_{k}P_{1}^{\left(k\right)}\left(X_{1}|a_{1}\right)$$

and

(7) $$\int P\left(X_{1},X_{2}|a_{1},a_{2}\right)dX_{1}=\sum_{k}P_{k}P_{2}^{\left(k\right)}\left(X_{2}|a_{2}\right).$$

For the entangled probability distributions, we apply the probability distribution $\Pi (X_{1}|a_{1})$ as the integral (6)

(8) $$\int P\left(X_{1},X_{2}|a_{1},a_{2}\right)dX_{2}=\Pi \left(X_{1}|a_{1}\right),$$

and it cannot be presented as a convex sum as in (6).

The proof of the independence of the integral (8) on the parameter $a_{2}$ is analogous to the proof of (1), where one uses the Bayes formula for the function of two random variables, $X_{1}$ and $X_{2}$, and two conditions described by the parameters $a_{1}$ and $a_{2}$, which can be multi-component parameters.

## 3. Examples of the Entangled Probability Distributions

The entangled probability distribution can be related to probability distributions realized by using the superposition principle of quantum state wave functions; for example, the superposition of Fock states resembling states of two–mode oscillators with the wave functions. We consider the very simple model of state $\psi_{+}\left(x_{1},x_{2}\right)$ of the form

(9) $$\psi_{+}\left(x_{1},x_{2}\right)=\frac{1}{\sqrt{2}}\left(\psi_{0}\left(x_{1}\right)\psi_{1}\left(x_{2}\right)+\psi_{1}\left(x_{1}\right)\psi_{0}\left(x_{2}\right)\right)=\frac{x_{1}+x_{2}}{\sqrt{\pi }}\exp\left(-\frac{x_{1}^{2}}{2}-\frac{x_{2}^{2}}{2}\right).$$

The function (9) is the superposition of wave functions of two-mode oscillators. The functions $|\psi_{0}\left(x_{1}\right)\rangle$ and $|\psi_{0}\left(x_{2}\right)\rangle$ are ground states of the first and second oscillators, i.e.,

(10) $$\psi_{0}\left(x_{1}\right)=\frac{e^{-\frac{x_{1}^{2}}{2}}}{\pi^{1/4}},\;\psi_{0}\left(x_{2}\right)=\frac{e^{-\frac{x_{2}^{2}}{2}}}{\pi^{1/4}},$$

and the function $\psi_{1}\left(x_{1}\right)$ is the first excited state of the first oscillator, and $\psi_{1}\left(x_{2}\right)$ is the first excited state of the second oscillator, i.e.,

(11) $$\psi_{1}\left(x_{1}\right)=\frac{\sqrt{2}x_{1}}{\pi^{1/4}}e^{-\frac{x_{1}^{2}}{2}},\;\psi_{1}\left(x_{2}\right)=\frac{\sqrt{2}x_{2}}{\pi^{1/4}}e^{-\frac{x_{2}^{2}}{2}}.$$

One can extend the construction of the superposition function (9) by following the superposition states of the oscillators studied in the literature, which are even and odd states [49]. The state (9) is the even state, i.e., $\psi_{+}\left(-x_{1},-x_{2}\right)=\psi_{+}\left(x_{1},x_{2}\right)$ and one can consider an odd state, which reads

(12) $$\psi_{-}\left(x_{1},x_{2}\right)=\frac{1}{\sqrt{2}}\left(\psi_{0}\left(x_{1}\right)\psi_{1}\left(x_{2}\right)-\psi_{1}\left(x_{1}\right)\psi_{0}\left(x_{2}\right)\right)=\frac{x_{2}-x_{1}}{\sqrt{\pi }}\exp\left(-\frac{x_{1}^{2}}{2}-\frac{x_{2}^{2}}{2}\right).$$

This state is the odd state, i.e., $\psi_{-}\left(-x_{1},-x_{2}\right)=-\psi_{-}\left(x_{1},x_{2}\right)$, which is the analog of odd coherent states (odd Schrödinger cat states). The more general superposition state $\psi_{b}\left(x_{1},x_{2}\right)$ can be given by the following construction

(13) $$\begin{array}{ccc} & & \psi_{b}\left(x_{1},x_{2}\right)=\frac{1}{\sqrt{2}}\left(\psi_{0}\left(x_{1}\right)\psi_{1}\left(x_{2}\right)+e^{ib}\psi_{1}\left(x_{1}\right)\psi_{0}\left(x_{2}\right)\right)\\ & & =\frac{1}{\sqrt{\pi }}\left(x_{2}+e^{ib}x_{1}\right)\exp\left(-\frac{x_{1}^{2}}{2}-\frac{x_{2}^{2}}{2}\right). \end{array}$$

An example of a simple separable state is a state that is not a superposition state, for instance,

(14) $$\psi_{s}\left(x_{1},x_{2}\right)=\psi_{0}\left(x_{1}\right)\psi_{1}\left(x_{2}\right).$$

Using the relationship between the symplectic tomogram and the wave function [50]

(15) $$\begin{array}{ccc} & & w(X_{1},X_{2}|\mu_{1},\mu_{2},\nu_{1},\nu_{2})=\\ & & \frac{1}{4\pi^{2}|\nu_{1}\left|\right|\nu_{2}|}{\left|\int \psi \left(x_{1},x_{2}\right)\exp\left(\frac{i\mu_{1}}{2\nu_{1}}x_{1}^{2}+\frac{i\mu_{2}}{2\nu_{2}}x_{2}^{2}-\frac{iX_{1}x_{1}}{2\nu_{1}}-\frac{iX_{2}x_{2}}{2\nu_{2}}\right)dx_{1}dx_{2}\right|}^{2}, \end{array}$$

one can obtain the explicit form of the conditional probability distribution $w_{+}\left(X_{1},X_{2}|\mu_{1},\nu_{1},\mu_{2},\nu_{2}\right)$ for the even state (9), i.e.,

(16) $$\begin{array}{ccc} & & w_{+}\left(X_{1},X_{2}|\mu_{1},\nu_{1},\mu_{2},\nu_{2}\right)=\frac{\left(\nu_{2}^{2}+\mu_{2}^{2}\right)X_{1}^{2}+2\left(\nu_{1}\nu_{2}+\mu_{1}\mu_{2}\right)X_{1}X_{2}+\left(\nu_{1}^{2}+\mu_{1}^{2}\right)X_{2}^{2}}{\pi {\left(\nu_{1}^{2}+\mu_{1}^{2}\right)}^{3/2}{\left(\nu_{2}^{2}+\mu_{2}^{2}\right)}^{3/2}}\\ & & \times \exp\left[-\frac{X_{1}^{2}}{\mu_{1}^{2}+\nu_{1}^{2}}-\frac{X_{2}^{2}}{\mu_{2}^{2}+\nu_{2}^{2}}\right]. \end{array}$$

For the particular case where $\nu_{1}=\nu_{2}=1,\mu_{1}=\mu_{2}=0,$ one gets

(17) $$w_{+}\left(X_{1},X_{2}|\mu_{1}=0,\nu_{1}=1,\mu_{2}=0,\nu_{2}=1\right)=\frac{1}{\pi }{\left(X_{1}+X_{2}\right)}^{2}\exp\left(-X_{1}^{2}-X_{2}^{2}\right).$$

One can check that the function $w_{+}\left(X_{1},X_{2}|\mu_{1},\nu_{1},\mu_{2},\nu_{2}\right)$ (17) satisfies the condition

(18) $$\int \int w_{+}\left(X_{1},X_{2},|\mu_{1},\nu_{1},\mu_{2},\nu_{2}\right)dX_{1}dX_{2}=1.$$

As we know, this probability distribution function corresponding to superposition of the wave functions (9) determines the quantum state, which is the entangled state. Due to this, we call this probability distribution the entangled probability distribution. In quantum mechanics, the wave functions of two–mode oscillators, which are obtained by means of superposition of two different wave functions, are entangled pure states. In connection with this, the tomographic probability distribution is described by the probability distribution function (16), and it cannot be represented in the form of Equation (3). On the other hand, it can be represented by the integral

(19) $$w_{+}\left(X_{1}|\mu_{1},\nu_{1}\right)=\int w_{+}\left(X_{1},X_{2}|\mu_{1},\nu_{1},\mu_{2},\nu_{2}\right)dX_{2}=\frac{\exp\left(-\frac{X_{1}^{2}}{\mu_{1}^{2}+\nu_{1}^{2}}\right)}{\sqrt{\pi \left(\mu_{1}^{2}+\nu_{1}^{2}\right)}}\left[\frac{1}{2}+\frac{X_{1}^{2}}{\mu_{1}^{2}+\nu_{1}^{2}}\right].$$

One can check that

(20) $$\int w_{+}\left(X_{1}|\mu_{1},\nu_{1}\right)dX_{1}=1.$$

The function $w_{+}\left(X_{1}|\mu_{1},\nu_{1}\right)$ (19) is a marginal conditional probability distribution of position $X_{1}$, which is the position of the first oscillator, and the conditions are labeled by the real parameters $\mu_{1}$ and $\nu_{1}$. Moreover, if we repeat analogous calculations for the second oscillator, we get

(21) $$w_{+}\left(X_{2}|\mu_{2},\nu_{2}\right)=\int w_{+}\left(X_{1},X_{2}|\mu_{1},\nu_{1},\mu_{2},\nu_{2}\right)dX_{1}=\frac{\exp\left(-\frac{X_{2}^{2}}{\mu_{2}^{2}+\nu_{2}^{2}}\right)}{\sqrt{\pi \left(\mu_{2}^{2}+\nu_{2}^{2}\right)}}\left[\frac{1}{2}+\frac{X_{2}^{2}}{\mu_{2}^{2}+\nu_{2}^{2}}\right].$$

One can check that

(22) $$\int w_{+}\left(X_{2}|\mu_{2},\nu_{2}\right)dX_{2}=1.$$

The function $w_{+}\left(X_{2}|\mu_{2},\nu_{2}\right)$ is a marginal conditional probability distribution of position $X_{2}$, which is the position of the second oscillator, and the conditions are labeled by the real parameters $\mu_{2}$ and $\nu_{2}$.

The function (16) is the probability distribution function; it has the form of the sum of three functions that contain products of Gaussian functions and different terms of position products of $X_{1}$ and $X_{2}$. The two terms are the probability distribution functions. The third term that is obtained from the integral (15) is not a probability distribution function, but when added to the two terms mentioned above it gives the probability distribution function (tomographic probability distribution).

An analogous procedure can be followed in the case of the odd states, and one gets the explicit form of the conditional probability distribution $w_{-}\left(X_{1},X_{2}|\mu_{1},\nu_{1},\mu_{2},\nu_{2}\right)$ for the odd state (12), i.e.,

(23) $$\begin{array}{ccc} & & w_{-}\left(X_{1},X_{2}|\mu_{1},\nu_{1},\mu_{2},\nu_{2}\right)=\frac{\left(\nu_{2}^{2}+\mu_{2}^{2}\right)X_{1}^{2}-2\left(\nu_{1}\nu_{2}+\mu_{1}\mu_{2}\right)X_{1}X_{2}+\left(\nu_{1}^{2}+\mu_{1}^{2}\right)X_{2}^{2}}{\pi {\left(\nu_{1}^{2}+\mu_{1}^{2}\right)}^{3/2}{\left(\nu_{2}^{2}+\mu_{2}^{2}\right)}^{3/2}}\\ & & \times \exp\left[-\frac{X_{1}^{2}}{\mu_{1}^{2}+\nu_{1}^{2}}-\frac{X_{2}^{2}}{\mu_{2}^{2}+\nu_{2}^{2}}\right]. \end{array}$$

Additionally, in the case of state with the phase (13), we obtain the explicit form of the conditional probability distribution

(24) $$\begin{array}{ccc} & & w_{b}\left(X_{1},X_{2}|\mu_{1},\nu_{1},\mu_{2},\nu_{2}\right)=\\ & & \frac{\left(\nu_{2}^{2}+\mu_{2}^{2}\right)X_{1}^{2}+2X_{1}X_{2}\cos\left[b-\arccos\left(\left(\mu_{1}^{2}+\nu_{1}^{2}\right)\left(\mu_{2}^{2}+\nu_{2}^{2}\right)\right)\right]+\left(\nu_{1}^{2}+\mu_{1}^{2}\right)X_{2}^{2}}{\pi {\left(\nu_{1}^{2}+\mu_{1}^{2}\right)}^{3/2}{\left(\nu_{2}^{2}+\mu_{2}^{2}\right)}^{3/2}}\\ & & \times \exp\left[-\frac{X_{1}^{2}}{\mu_{1}^{2}+\nu_{1}^{2}}-\frac{X_{2}^{2}}{\mu_{2}^{2}+\nu_{2}^{2}}\right]. \end{array}$$

For the separable state (14), the tomographic probability distribution given by the Formula (15) reads

(25) $$w_{0,1}\left(X_{1},X_{2}|\mu_{1},\nu_{1},\mu_{2},\nu_{2}\right)=\frac{2X_{2}^{2}}{\sqrt{\pi^{2}\left(\mu_{1}^{2}+\nu_{1}^{2}\right)}{\left(\mu_{2}^{2}+\nu_{2}^{2}\right)}^{3/2}}\exp\left(-\frac{X_{1}^{2}}{\mu_{1}^{2}+\nu_{1}^{2}}-\frac{X_{2}^{2}}{\mu_{2}^{2}+\nu_{2}^{2}}\right).$$

The probability distribution (25) has the form of the product of the probability distributions of each mode state $w_{0}\left(X_{1}|\mu_{1},\nu_{1}\right)$ and $w_{1}\left(X_{2}|\mu_{2},\nu_{2}\right)$.

Our assertion is as follows: The structure of an entangled probability distribution in the general case always has the form of the sum of two terms, namely, the first term is the convex sum of the products of the probability distributions, and the second term is the sum of the products of the terms that are not probabilities. However, when summed, these two terms lead to an entangled probability distribution. This entangled probability distribution has all the properties of the conventional probability distribution.

## 4. Evolution of States in Different Representations

Let us recount the description of quantum state dynamics in the Hilbert space H, where the pure quantum state is associated with the state vector |ψ〉[2], and the other states, including the pure states, are also described by the density operators $\hat{\rho }$ [3,4] acting on the vectors in the Hilbert space H. The dynamics of the states are described by the Schrödinger equation (ℏ=1)

(26) $$i\frac{\partial |\psi (t)\rangle }{\partial t}=\hat{H}|\psi \left(t\right)\rangle ,$$

where $\hat{H}$ is the system Hermitian Hamiltonian $\left(\hat{H}={\hat{H}}^{†}\right)$.

For the time-independent Hamiltonian, the state vector |ψ〉 evolves by means of the evolution operator $\hat{u}\left(t\right)=\exp\left(-i\hat{H}t\right)$ of the form

(27) $$|\psi \left(t\right)\rangle =\hat{u}\left(t\right)|\psi \left(0\right)\rangle ,\;\hat{u}\left(0\right)=\hat{1}.$$

For the pure state with the state vector |ψ(t)〉, the density operator is given by the formula $\hat{\rho }\left(t\right)=\left|\psi \left(t\right)\rangle \langle \psi \left(t\right)\right|$, and the Schrödinger Equation (27) provides the equation for the density operator of the form (the von Neumann equation)

(28) $$i\frac{\partial (|\psi (t)\rangle \langle \psi (t)|)}{\partial t}=\hat{H}\left|\psi \left(t\right)\rangle \langle \psi \left(t\right)\right|-\left|\psi \left(t\right)\rangle \langle \psi \left(t\right)\right|\hat{H}.$$

This equation is also valid for mixed states with the Hermitian density operator $\hat{\rho }\left(t\right)=\sum_{k}\lambda_{k}|\psi_{k}\left(t\right)\rangle \langle \psi_{k}\left(t\right)|$. Here, the parameters $\lambda_{k}$ are probabilities describing mixed states. The equation can be given in the following form

(29) $$\frac{\partial \hat{\rho }\left(t\right)}{\partial t}+i\left[\hat{H},\rho \left(t\right)\right]=0.$$

The solution of this equation, corresponding to the solution of Equation (27), reads

(30) $$\hat{\rho }\left(t\right)=\hat{u}\left(t\right)\hat{\rho }\left(0\right){\hat{u}}^{†}\left(t\right).$$

The operators such as position $\hat{q}$ and momentum $\hat{p}$ operators in the Heisenberg representation, namely, ${\hat{q}}_{H}\left(t\right)$ and ${\hat{p}}_{H}\left(t\right)$, are given as follows

(31) $${\hat{q}}_{H}\left(t\right)={\hat{u}}^{†}\left(t\right)\hat{q}\hat{u}\left(t\right),\;{\hat{p}}_{H}\left(t\right)={\hat{u}}^{†}\left(t\right)\hat{p}\hat{u}\left(t\right).$$

The integrals of motion ${\hat{q}}_{0}\left(t\right)$ and ${\hat{p}}_{0}\left(t\right)$, which have the initial values ${\hat{q}}_{0}\left(t=0\right)=\hat{q}$ and ${\hat{p}}_{0}\left(t=0\right)=\hat{p}$ and satisfy Equation (29), are connected with the Heisenberg position and momentum operators for time-independent Hamiltonian by the relationship

(32) $${\hat{q}}_{0}\left(-t\right)={\hat{q}}_{H}\left(t\right),\;{\hat{p}}_{0}\left(-t\right)={\hat{p}}_{H}\left(t\right).$$

The stationary states of a system $|\psi_{E}\left(t\right)\rangle$ satisfying the Schrödinger equation (26) have the form

(33) $$|\psi_{E}\left(t\right)\rangle =\hat{u}\left(t\right)|\psi_{E}\left(0\right)\rangle =\exp\left(-iEt\right)|\psi_{E}\left(0\right)\rangle ,$$

where the vector $|\psi_{E}\left(0\right)\rangle$ is the eigenvector of the Hamiltonian operator, i.e.,

(34) $$\hat{H}|\psi_{E}\left(0\right)\rangle =E|\psi_{E}\left(0\right)\rangle .$$

The eigenvalue parameter *E* describes the energy level of the system. The superposition principle of quantum states means that the vector |ψ(t)〉 of the form

(35) $$|\psi \left(t\right)\rangle =\sum_{k}C_{k}|\psi_{E_{k}}\left(t\right)\rangle ,$$

where $C_{k}$ are complex numbers, is the solution of the Schrödinger equation (26). Furthermore, it means that the density operator $\hat{\rho }\left(t\right)$ of the form

(36) $$\hat{\rho }\left(t\right)=\sum_{k}\sum_{k^{\prime }}C_{k}C_{k^{\prime }}^{*}|\psi_{E_{k}}\left(t\right)\rangle \langle \psi_{E_{k^{\prime }}}\left(t\right)|$$

is the solution of the von Neumann equation (29). Moreover, it means that due to Equations (33) and (34), we have

(37) $$\hat{\rho }\left(t\right)=\sum_{k}\sum_{k^{\prime }}C_{k}C_{k^{\prime }}^{*}\exp\left(i(E_{k}-E_{k^{\prime }})t\right)|\psi_{E_{k}}\left(t\right)\rangle \langle \psi_{E_{k^{\prime }}}\left(t\right)|,$$

or

(38) $$\hat{\rho }\left(t\right)=\sum_{k}|C_{k}{|}^{2}|\psi_{E_{k}}\left(0\right)\rangle \langle \psi_{E_{k}}\left(0\right)|+\sum_{k}\sum_{k^{\prime }\neq k}C_{k}C_{k^{\prime }}^{*}\exp\left(i(E_{k}-E_{k^{\prime }})t\right)|\psi_{E_{k}}\left(0\right)\rangle \langle \psi_{E_{k^{\prime }}}\left(0\right)|.$$

The dynamics of the state density operator are determined for all the states, which can be represented as superpositions of energy level states by Formula (38) since the vectors $|\psi_{E_{k}}\left(t\right)\rangle$ form the complete system of vectors in the Hilbert space H.

## 5. Quantum States Definition

Now we consider different representations of quantum states using the formalism of quantizer–dequantizer operators $\hat{D}\left(\vec{x}\right)$ and $\hat{U}\left(\vec{x}\right)$[51], where $\vec{x}$ is a set of parameters $\left(x_{1},x_{2},\ldots ,x_{n}\right)$ such that the density operators $\hat{\rho }$ can be mapped onto the set of functions $f_{\rho }\left(\vec{x}\right)$, which are named symbols of operators, i.e.,

(39) $$f_{\rho }\left(\vec{x}\right)=\mathrm{Tr}\hat{\rho }\hat{U}\left(\vec{x}\right).$$

The operator $\hat{U}\left(\vec{x}\right)$ is a dequantizer operator. It maps the operator on its symbol. The density operator can be reconstructed from the symbol of the density operator with the help of the inverse transform

(40) $$\hat{\rho }=\int f_{\rho }\left(\vec{x}\right)\hat{D}\left(\vec{x}\right)d\vec{x}.$$

The operator $\hat{D}\left(\vec{x}\right)$ is a quantizer operator. All the state representations, including the Wigner function [5], Husimi function [45], and the Glauber–Sudarshan function [46,47], and corresponding symbols of other operators are formulated using corresponding quantizer–dequantizer operators. Thus, the quantum mechanics can be formulated using the formalism of operators acting in the Hilbert space or their symbols that contain the same information on quantum states. One can transform the quantum mechanics formalism and obtain equations (differential or integral) for the density operator symbols. An important novelty is that the possibility of describing quantum states by conventional probability distributions exists [48,52].

All known functions that are quasiprobability distributions and describe the states of quantum systems can be obtained using various pairs of the quantizer operator $\hat{D}\left(\vec{x}\right)$ and the dequantizer operator $\hat{U}\left(\vec{x}\right)$, where $\vec{x}=x_{1},x_{2},\ldots x_{n}$. These operators make it possible to map any operator $\hat{A}$ acting in a Hilbert space, where the position operator $\hat{q}$ and momentum operator $\hat{p}$ act on the function $f_{A}\left(\vec{x}\right)$, which is called the symbol of the operator $\hat{A}$, using the following general mapping of the operators $\hat{A}\to f_{A}\left(\vec{x}\right)$ to functions, i.e.,

(41) $$f_{A}\left(\vec{x}\right)=\mathrm{Tr}\left(\hat{A}\hat{U}\left(\vec{x}\right)\right).$$

The operator $\hat{A}$ can be reconstructed from its symbol $f_{A}\left(\vec{x}\right)$ using the inverse transform $f_{A}\left(\vec{x}\right)\to \hat{A}$, i.e.,

(42) $$\hat{A}=\int f_{A}\left(\vec{x}\right)\hat{D}\left(\vec{x}\right)d\vec{x}.$$

So, the operator $\hat{A}$ can be reconstructed from its symbol if the quantizer operator $\hat{D}\left(\vec{x}\right)$ and the symbol of operator $f_{A}\left(\vec{x}\right)$ are known. The map given by Equations (41) and (42) provides the possibility to introduce the star product of functions $f_{A}\left(\vec{x}\right)$ and $f_{B}\left(\vec{x}\right)$, which are symbols of operators $\hat{A}$ and $\hat{B}$. The symbol of operator $\hat{A}\hat{B}$, which is product of operators $\hat{A}$ and $\hat{B}$, is

(43) $$f_{AB}\left(\vec{x}\right)=\mathrm{Tr}\left(\hat{A}\hat{B}\hat{U}\left(\vec{x}\right)\right).$$

We can present the star product of the functions $f_{A}\left(\vec{x}\right)$ and $f_{B}\left(\vec{x}\right)$

(44) $$\left(f_{A}⋆f_{B}\right)\left(\vec{x}\right)=f_{AB}\left(\vec{x}\right)$$

using the relationships (41)–(43) in the integral form

(45) $$\left(f_{A}⋆f_{B}\right)\left(\vec{x}\right)=\int f_{A}\left({\vec{x}}_{1}\right)f_{B}\left({\vec{x}}_{2}\right)K\left({\vec{x}}_{1},{\vec{x}}_{2},\vec{x}\right)d{\vec{x}}_{1}d{\vec{x}}_{2}.$$

The kernel $K({\vec{x}}_{1},{\vec{x}}_{2},\vec{x})$ is expressed in terms of the quantizer and dequantizer operators

(46) $$K\left({\vec{x}}_{1},{\vec{x}}_{2},\vec{x}\right)=\mathrm{Tr}\left(\hat{D}\left({\vec{x}}_{1}\right)\hat{D}\left({\vec{x}}_{2}\right)\hat{U}\left(\vec{x}\right)\right).$$

The associativity condition for the product of operators, i.e., $\left(\left(\hat{A}\hat{B}\right)\hat{C})=(\hat{A}\left(\hat{B}\hat{C}\right)\right)$ causes the star product of operator symbols to be associative as well. The formalism of quantizer–dequantizer operators can be used to determine the evolution equation for the symbols of the density operators. The von Neumann equation for the oscillator density operator $\hat{\rho }\left(t\right)$ is written as (we use m=ω=ℏ=1)

(47) $$\frac{\partial \hat{\rho }}{\partial t}+i\left(\hat{H}\left(t\right)\hat{\rho }\left(t\right)-\hat{\rho }\left(t\right)\hat{H}\left(t\right)\right)=0.$$

If we introduce the symbol $f_{\rho }\left(\vec{x},t\right)$ of the density operator $\hat{\rho }\left(t\right)$ and the symbol $f_{H}\left(\vec{x},t\right)$ of the Hamiltonian operator $\hat{H}\left(t\right)$, using an arbitrary pair of quantizer and dequantizer operators, then Equation (47) becomes

(48) $$\frac{\partial f_{\rho }\left(\vec{x},t\right)}{\partial t}+i\left(f_{H}⋆f_{\rho }-f_{\rho }⋆f_{H}\right)\left(\vec{x},t\right)=0.$$

The equation for the evolution of the density operator symbol for the given Hamiltonian $\hat{H}\left(t\right)$ has the general form of an integral equation

(49) $$\frac{\partial f_{\rho }\left(\vec{x},t\right)}{\partial t}+i\int \left(f_{H}\left({\vec{x}}_{1},t\right)f_{\rho }\left({\vec{x}}_{2},t\right)-f_{\rho }\left({\vec{x}}_{1},t\right)f_{H}\left({\vec{x}}_{2},t\right)\right)K\left({\vec{x}}_{1},{\vec{x}}_{2},\vec{x}\right)d{\vec{x}}_{1}d{\vec{x}}_{2}=0.$$

Here, the symbol of the Hamiltonian $f_{H}\left({\vec{x}}_{1},t\right)=\mathrm{Tr}(\hat{H}\left(t\right)\hat{U}\left({\vec{x}}_{1}\right))$ and the symbol of density operator $f_{\rho }\left({\vec{x}}_{2},t\right)=\mathrm{Tr}(\hat{\rho }\left(t\right)\hat{U}\left({\vec{x}}_{2}\right)).$ Using (39), (46), and (49), one obtains

(50) $$\begin{array}{ccc} & & \frac{\partial f_{\rho }\left(\vec{x},t\right)}{\partial t}+i\int \left[\mathrm{Tr}(\hat{H}\left(t\right)\hat{U}\left({\vec{x}}_{1}\right))\mathrm{Tr}(\hat{\rho }\left(t\right)\hat{U}\left({\vec{x}}_{2}\right))-\mathrm{Tr}(\hat{\rho }\left(t\right)\hat{U}\left({\vec{x}}_{1}\right))\mathrm{Tr}(\hat{H}\left(t\right)\hat{U}\left({\vec{x}}_{2}\right))\right]\\ & & \times \mathrm{Tr}(\hat{D}\left({\vec{x}}_{1}\right)\hat{D}\left({\vec{x}}_{2}\right)\hat{U}\left(\vec{x}\right))d{\vec{x}}_{1}d{\vec{x}}_{2}=0. \end{array}$$

Equation (50) can be written in the form of a kinetic equation for a probability distribution function

(51) $$\frac{\partial f_{\rho }\left(\vec{x},t\right)}{\partial t}+i\int f_{\rho }\left({\vec{x}}_{2},t\right)K\left(\vec{x},{\vec{x}}_{2},t\right)d{\vec{x}}_{2}=0,$$

where

(52) $$K\left(\vec{x},{\vec{x}}_{2},t\right)=\int \left(K\left({\vec{x}}_{1},{\vec{x}}_{2},x,t\right)-K\left({\vec{x}}_{2},{\vec{x}}_{1},x,t\right)\right)f_{H}\left({\vec{x}}_{1},t\right)d{\vec{x}}_{1},$$

and the symbol of density operator $f_{\rho }\left(\vec{x},t\right)$ is a probability distribution. For the symplectic tomogram, the inverse quantum Radon transform reads [53,54]

(53) $$\hat{\rho }=\frac{1}{2\pi }\int w\left(X|\mu ,\nu \right)\exp\left(i(X\hat{1}-\mu \hat{q}-\nu \hat{p})\right)dXd\mu d\nu .$$

This means that the quantizer operator for the symplectic tomography method has the form

(54) $$\hat{D}\left(X|\mu ,\nu \right)=\frac{1}{2\pi }\exp\left(i(X\hat{1}-\mu \hat{q}-\nu \hat{p})\right).$$

Thus, we have $\vec{x}=X,\mu ,\nu$, and the dequantizer reads

(55) $$\hat{U}\left(X|\mu ,\nu \right)=\delta \left(i(X\hat{1}-\mu \hat{q}-\nu \hat{p})\right).$$

The existence of (55) provides the possibility to map the density operator $\hat{\rho }$ (applying Formula (39)) onto the function (tomogram of the state). The existence of quantizer (54) provides the possibility to reconstruct the density operator $\hat{\rho }$ using the tomogram of the quantum state. Such pairs of quantizer–dequantizer operators exist for all the other quantum systems, including the two-mode usual and inverted oscillators discussed in this paper.

The kernel describing the star product of the operators in the symplectic tomography is expressed as follows

(56) $$\begin{array}{ccc} & & K\left(X_{1},\mu_{1},\nu_{1},X_{2},\mu_{2},\nu_{2},X,\mu ,\nu \right)=\frac{1}{4\pi^{2}}\mathrm{Tr}\left[\exp\left(i(X_{1}\hat{1}-\mu_{1}\hat{q}-\nu_{1}\hat{p})\right)\right.\\ & & \times \left.\exp\left(i(X_{2}\hat{1}-\mu_{2}\hat{q}-\nu_{2}\hat{p})\right)\delta \left(i(X\hat{1}-\mu \hat{q}-\nu \hat{p})\right)\right]. \end{array}$$

In an explicit form it reads

(57) $$\begin{array}{ccc} & & K\left(X_{1},\mu_{1},\nu_{1},X_{2},\mu_{2},\nu_{2},X,\mu ,\nu \right)=\frac{1}{4\pi^{2}}\delta \left(\mu \left(\nu_{1}+\nu_{2}\right)-\nu \left(\mu_{1}+\mu_{2}\right)\right)\\ & & \times \exp\left(\frac{i}{2}\left(\nu_{1}\mu_{2}-\nu_{2}\mu_{1}+2X_{1}+2X_{2}-2\frac{\nu_{1}+\nu_{2}}{\nu }X\right)\right). \end{array}$$

In the case of a harmonic oscillator in the tomographic probability representation, the symbol of density operator $\hat{\rho }\left(t\right)$ is given by the probability distribution function, $(\vec{x}=X,\mu ,\nu ),$

(58) $$w_{\rho }\left(X|\mu ,\nu ,t\right)=f_{\rho }\left(\vec{x},t\right)=\mathrm{Tr}\hat{\rho }\left(t\right)\delta \left(X\hat{1}-\mu \hat{q}-\nu \hat{p}\right),$$

The Hamiltonian $\hat{H}$ can be mapped onto its symbol

(59) $$f_{\hat{H}}\left(X,\mu ,\nu \right)=\mathrm{Tr}\left(\hat{H}\delta \left(X\hat{1}-\mu \hat{q}-\nu \hat{p}\right)\right).$$

The symplectic tomogram (58) is the symbol of the density operator $\hat{\rho }$, and it is the probability distribution of position *X* [13] depending on extra parameters determining the reference frame in the phase space where the position *X* is measured. For symplectic tomography, the integral linear Equation (51) has the form

(60) $$\frac{\partial w_{\rho }\left(X|\mu ,\nu ,t\right)}{\partial t}+i\int w_{\rho }\left(X_{2}|\mu_{2},\nu_{2},t\right)K\left(X,\mu ,\nu ,X_{2},\mu_{2},\nu_{2},t\right)dX_{2}d\mu_{2}d\nu_{2}=0.$$

Here,

(61) $$\begin{array}{ccc} & & K(X,\mu ,\nu ,X_{2},\mu_{2},\nu_{2},t)=\\ & & \int \left[K\left(X_{1},\mu_{1},\nu_{1},X_{2},\mu_{2},\nu_{2},t\right)-K\left(X_{2},\mu_{2},\nu_{2},X_{1},\mu_{1},\nu_{1},t\right)\right]f_{H}\left(X_{1},\mu_{1},\nu_{1},t\right)dX_{1}d\mu_{1}d\nu_{1}. \end{array}$$

The product of operators $\hat{A}\cdot \hat{B}$ is mapped onto the star product of their symbols

(62) $$\left(\hat{A}\hat{B}\right)↔\left(A⋆B\right)\left(X,\mu ,\nu \right)=\mathrm{Tr}\left(\hat{A}\hat{B}\delta \left(X\hat{1}-\mu \hat{q}-\nu \hat{p}\right)\right)$$

with the kernel of the star product defined by means of the expression

(63) $$\begin{array}{ccc} & & (A⋆B)(X,\mu ,\nu )=\\ & & \int A\left(X_{1},\mu_{1}\nu_{1}\right)B\left(X_{2},\mu_{2},\nu_{2}\right)K\left(X_{1},\mu_{1}\nu_{1},X_{2},\mu_{2},\nu_{2},X,\mu ,\nu \right)dX_{1}dX_{2}d\mu_{1}d\mu_{2}d\nu_{1}d\nu_{2}. \end{array}$$

This formula is the application of general Formula (46) for the kernel of the star product of the symbols. This formula can be used to study the entanglement phenomena of states that are superpositions of two-mode oscillator states.

## 6. Symplectic Tomography of Oscillators

One can calculate the tomographic probability distribution w(X|μ,ν), called the symplectic tomogram of the state with density operator ${\hat{\rho }}_{|\psi \rangle }=\left|\psi \rangle \langle \psi \right|$, using the formula analogous to (15) expressed in terms of wave function ψ(y) of the pure state in position representation, which reads [50]

(64) $$w_{|\psi \rangle }\left(X|\mu ,\nu \right)=\frac{1}{2\pi |\nu |}{\left|\int \psi \left(y\right)\exp\left(\frac{i\mu }{2\nu }y^{2}-\frac{iXy}{\nu }\right)dy\right|}^{2}.$$

The function is non-negative and satisfies the normalization condition

(65) $$\int w_{|\psi \rangle }\left(X|\mu ,\nu \right)dX=1.$$

The physical meaning of the real parameters μ and ν is that they, due to using $\delta (X\hat{1}-\mu \hat{q}-\nu \hat{p})$ to determine the dequantizer $\hat{U}\left(x\right)$ as a delta function, describe the axes of reference frames in the phase space of position $\hat{q}$ and momentum $\hat{p}$, where the position $X\hat{1}=\mu \hat{q}+\nu \hat{p}$ is measured. Thus, the tomogram w(X|μ,ν) is the conditional probability distribution determining the density operator for the state. If μ=1,ν=0, it is the density matrix diagonal elements ρ(qq), and for μ=0,ν=1, the tomogram is the diagonal matrix element ρ(pp). This means that if one knows the probability distributions of position and momentum in all the reference frames in the phase space, the state (state density operator) is known.

In the case of two-mode oscillators, the relationship between the symplectic tomogram and wave function of the state is obtained by Equation (15). Using (15), one can obtain the symplectic tomogram of the ground state of two-mode oscillators in the explicit form

(66) $$w_{0}\left(X_{1},X_{2}|\mu_{1},\mu_{2},\nu_{1},\nu_{2}\right)=\frac{1}{\pi \sqrt{\mu_{1}^{2}+\nu_{1}^{2}}\sqrt{\mu_{2}^{2}+\nu_{2}^{2}}}\exp\left(-\frac{X_{1}^{2}}{\mu_{1}^{2}+\nu_{1}^{2}}-\frac{X_{2}^{2}}{\mu_{2}^{2}+\nu_{2}^{2}}\right),$$

and the tomogram of coherent state of two-mode oscillators in the Gaussian form

(67) $$w_{\alpha }\left(X_{1},X_{2}|\mu_{1},\mu_{2},\nu_{1},\nu_{2}\right)=\frac{1}{\pi \sqrt{\mu_{1}^{2}+\nu_{1}^{2}}\sqrt{\mu_{2}^{2}+\nu_{2}^{2}}}\exp\left(-\frac{{\left(X_{1}-{\bar{X}}_{1}\right)}^{2}}{\mu_{1}^{2}+\nu_{1}^{2}}-\frac{{\left(X_{2}-{\bar{X}}_{2}\right)}^{2}}{\mu_{2}^{2}+\nu_{2}^{2}}\right),$$

where X¯1=2μ1Reα+2ν1Imα, X¯2=2μ2Reα+2ν2Imα, and α is a complex number.

## 7. Dynamics of Operator Symbols for Quadratic Hamiltonians in Position and Momentum

Let us discuss the problem of finding the tomographic probability distribution evolution for the systems with Hamiltonians, which are quadratic forms in position and momentum operators. Such systems have integrals of motion that are linear in position and momentum operators. Furthermore, the position and momentum operators ${\hat{q}}_{H}\left(t\right)$ and ${\hat{p}}_{H}\left(t\right)$ are linear forms of the position $\hat{q}$ and momentum $\hat{p}$ operators with time-dependent coefficients [55]. Due to this, we can explicitly obtain the time dependence of the tomographic probability distributions describing the quantum states and corresponding to solutions of the Schrödinger equation for wave functions and the von Neumann equation for the density operators. The idea to obtain the solutions of these equations was formulated in [48,52]. It is based on the following observation: Since the system state tomogram is given by the symbol of the density operator $\hat{\rho }\left(t\right)$, i.e., (58), where the density operator evolution for the von Neumann equation is described by the evolution operator $\hat{u}\left(t\right)$, i.e., $\hat{\rho }\left(t\right)=\hat{u}\left(t\right)\hat{\rho }\left(0\right){\hat{u}}^{†}\left(t\right)$ the symbol of the density operator can be rewritten in the form $\mathrm{Tr}(\hat{\rho }\left(0\right)\delta (X-\mu {\hat{u}}^{†}\left(t\right)\hat{q}\hat{u}\left(t\right)-\nu {\hat{u}}^{†}\left(t\right)\hat{p}\hat{u}\left(t\right)).$ Here, ${\hat{u}}^{†}\left(t\right)\hat{q}\hat{u}\left(t\right)={\hat{q}}_{H}\left(t\right)$ and ${\hat{u}}^{†}\left(t\right)\hat{p}\hat{u}\left(t\right)={\hat{p}}_{H}\left(t\right)$ are the Heisenberg position and momentum operators. Such properties also occur in multi-mode systems with Hamiltonians, which are any quadratic forms in position and momentum operators; for example, for two-dimensional oscillators, both ordinary,

(68) $${\hat{H}}^{\left(1\right)}=\frac{{\hat{p}}_{1}^{2}}{2}+\frac{{\hat{p}}_{2}^{2}}{2}+\frac{{\hat{q}}_{2}^{2}}{2}+\frac{{\hat{q}}_{1}^{2}}{2},$$

and for two-dimensional oscillators, both inverted,

(69) $${\hat{H}}^{\left(2\right)}=\frac{{\hat{p}}_{1}^{2}}{2}+\frac{{\hat{p}}_{2}^{2}}{2}-\frac{{\hat{q}}_{2}^{2}}{2}-\frac{{\hat{q}}_{1}^{2}}{2}.$$

The Hamiltonian ${\hat{H}}_{2}$ corresponds to the motion of the inverted oscillator. For such Hamiltonians, one has time-dependent Heisenberg operators of position and momentum of the following forms: for the ordinary oscillator with the Hamiltonian (68)

(70) $${\hat{q}}_{H^{\left(1\right)};1}\left(t\right)=\cos t\cdot {\hat{q}}_{1}+\sin t\cdot {\hat{p}}_{1},\;{\hat{q}}_{H^{\left(1\right)};2}\left(t\right)=\cos t\cdot {\hat{q}}_{2}+\sin t\cdot {\hat{p}}_{2},$$

(71) $${\hat{p}}_{H^{\left(1\right)};1}\left(t\right)=-\sin t\cdot {\hat{q}}_{1}+\cos t\cdot {\hat{p}}_{1},\;{\hat{p}}_{H^{\left(1\right)};2}\left(t\right)=-\sin t\cdot {\hat{q}}_{2}+\cos t\cdot {\hat{p}}_{2};$$

and for the inverted oscillator with the Hamiltonian (69)

(72) $${\hat{q}}_{H^{\left(2\right)};1}\left(t\right)=\cosh t\cdot {\hat{q}}_{1}+\sinh t\cdot {\hat{p}}_{1},\;{\hat{q}}_{H^{\left(2\right)};2}\left(t\right)=\cosh t\cdot {\hat{q}}_{2}+\sinh t\cdot {\hat{p}}_{2},$$

(73) $${\hat{p}}_{H^{\left(2\right)};1}\left(t\right)=\sinh t\cdot {\hat{q}}_{1}+\cosh t\cdot {\hat{p}}_{1},\;{\hat{p}}_{H^{\left(2\right)};2}\left(t\right)=\sinh t\cdot {\hat{q}}_{2}+\cosh t\cdot {\hat{p}}_{2}.$$

Developed formalism provides the possibility to obtain the description of time evolution for all the multi-mode systems with time-dependent quadratic Hamiltonians. For such systems, the Heisenberg position and momentum operators are linear forms with time-dependent coefficients of usual positions and momenta.

## 8. Center-of-Mass Tomography

Let us introduce a dequantizer operator for the two-mode oscillator $\hat{U}\left(X_{1},X_{2},\mu_{1},\nu_{1},\mu_{1},\nu_{2}\right)$. Then, the symplectic tomogram reads

(74) $$w\left(X_{1},X_{2}|\mu_{1},\nu_{1},\mu_{2},\nu_{2}\right)=\mathrm{Tr}\left(\hat{\rho }\delta \left(X_{1}\hat{1}-\mu_{1}{\hat{q}}_{1}-\nu_{1}{\hat{p}}_{1}\right)\delta \left(X_{2}\hat{1}-\mu_{2}{\hat{q}}_{2}-\nu_{2}{\hat{p}}_{2}\right)\right).$$

The dequantizer operator $\hat{U}\left(\vec{x}\right)$ in the case of the symplectic tomogram is $\delta \left(X_{1}\hat{1}-\mu_{1}{\hat{q}}_{1}-\nu_{1}{\hat{p}}_{1}\right)\delta \left(X_{2}\hat{1}-\mu_{2}{\hat{q}}_{2}-\nu_{2}{\hat{p}}_{2}\right)$. The density operator can be reconstructed from the symplectic tomogram with the help of the quantizer operator $\hat{D}\left(X_{1},X_{2},\mu_{1},\mu_{2},\nu_{1},\nu_{2}\right)=\frac{1}{4\pi^{2}}\exp\left(iX_{1}\hat{1}-\mu_{1}{\hat{q}}_{1}-\nu_{1}{\hat{p}}_{1}\right)\exp\left(iX_{2}\hat{1}-\mu_{2}{\hat{q}}_{2}-\nu_{2}{\hat{p}}_{2}\right)$. Then, the symplectic tomogram of the first mode of oscillator is related to (74) as

(75) $$w\left(X_{1}|\mu_{1},\nu_{1}\right)=\int w\left(X_{1},X_{2}|\mu_{1},\nu_{1},\mu_{2},\nu_{2}\right)dX_{2}.$$

There is another type of tomography, named center-of-mass tomography. It was introduced in [56] and developed in [57,58]. In center-of-mass tomography, the state is determined by the center-of-mass tomogram. The center-of-mass tomogram is a symbol of the density operator

(76) $$w_{cm}\left(X|\mu_{1},\nu_{1},\mu_{2},\nu_{2}\right)=\mathrm{Tr}\left(\hat{\rho }\delta \left(X\hat{1}-\mu_{1}{\hat{q}}_{1}-\nu_{1}{\hat{p}}_{1}-\mu_{2}{\hat{q}}_{2}-\nu_{2}{\hat{p}}_{2}\right)\right).$$

The random variable *X*, which we named the center-of-mass coordinate, is measured in phase space in rotated and scaled reference frames, which are determined by parameters $\mu_{1},\nu_{1},\mu_{2},\nu_{2}$. The dequantizer operator in center-of-mass tomography is

(77) $$\hat{U}\left(X,\mu_{1},\nu_{1},\mu_{2},\nu_{2}\right)=\delta \left(X\hat{1}-\mu_{1}{\hat{q}}_{1}-\nu_{1}{\hat{p}}_{1}-\mu_{2}{\hat{q}}_{2}-\nu_{2}{\hat{p}}_{2}\right).$$

The density operator can be reconstructed from the center-of-mass tomogram with the help of the quantizer operator $\hat{D}\left(X,\mu_{1},\nu_{1},\mu_{1},\nu_{2}\right)$, i.e.,

(78) $$\hat{\rho }=\frac{1}{4\pi^{2}}\int w_{cm}\left(X|\mu_{1},\nu_{1},\mu_{2}.\nu_{2}\right)\exp\left(i\left(X\hat{1}-\mu_{1}\hat{q}-\nu_{1}{\hat{p}}_{1}-\mu_{2}{\hat{q}}_{2}-\nu_{2}{\hat{p}}_{2}\right)\right)dXd\mu_{1}d\nu_{1}d\mu_{2}d\nu_{2}.$$

The center-of-mass tomogram of odd and even coherent states is of the form

(79) wcm,α(X|μ1,ν1,μ2,ν2)=1πσN±2(α)exp−(X−2Reα1μ1−2Reα2μ2−2Imα1ν1−2Imα2ν2)2/σ±exp−2|α1|−2|α2|−(X−i2Imα1μ1−i2Imα2μ2+i2Reα1ν1+i2Reα2ν2)2/σ±exp−2|α1|−2|α2|−(X+i2Imα1μ1+i2Imα2μ2−i2Reα1ν1−i2Reα2ν2)2/σ+exp−(X+2Reα1μ1+2Reα2μ2+2Imα1ν1+2Imα2ν2)2/σ,

where $\sigma =\mu_{1}^{2}+\mu_{2}^{2}+\nu_{1}^{2}+\nu_{2}^{2}$ and $N_{\pm }^{2}\left(\alpha \right)=2\left(1\pm \exp(-2|\alpha_{1}{|}^{2}-2|\alpha_{2}{|}^{2})\right)$. These tomograms (79) determine the nonclassical even and odd coherent states in the probability representation of quantum mechanics.

Following the method described in Section 7, we obtain the time-dependent center-of-mass tomogram of Schrödinger cat states. This means that in Formula (79), we have to replace $\mu_{1},\nu_{1},\mu_{2},\nu_{2}$ with time-dependent Heisenberg parameters in the case of evolution with the Hamiltonian of ordinary oscillator (68) of the form

(80) $$\begin{array}{ccc} & & \mu_{H^{\left(1\right)};1}=\mu_{1}\cos t-\nu_{1}\sin t,\;\mu_{H^{\left(1\right)};2}=\mu_{2}\cos t-\nu_{2}\sin t,\\ & & \nu_{H^{\left(1\right)};1}=\mu_{1}\sin t+\nu_{1}\cos t,\;\nu_{H^{\left(1\right)};2}=\mu_{2}\sin t+\nu_{2}\cos t. \end{array}$$

So, for the initial center-of-mass tomogram of odd and even states given by (79) after evolution with the Hamiltonian (68), one obtains the explicit expression

(81) wcm,α(X|μ1,ν1,μ2,ν2,t)=1πσN±2(α)exp−(X−2Reα1(μ1cost−ν1sint)−2Reα2(μ2cost−ν2sint)−2Imα1(μ1sint+ν1cost)−2Imα2(μ2sint+ν2cost))2/σ±exp−2|α1|−2|α2|−(X−i2Imα1(μ1cost−ν1sint)−i2Imα2(μ2cost−ν2sint)+i2Reα1(μ1sint+ν1cost)+i2Reα2(μ2sint+ν2cost))2/σ±exp−2|α1|−2|α2|−(X+i2Imα1(μ1cost−ν1sint)+i2Imα2(μ2cost−ν2sint)−i2Reα1(μ1sint+ν1cost)−i2Reα2(μ2sint+ν2cost))2/σ+exp−(X+2Reα1(μ1cost−ν1sint)+2Reα2(μ2cost−ν2sint)+2Imα1(μ1sint+ν1cost)+2Imα2(μ2sint+ν2cost))2/σ,

where $\sigma =\mu_{1}^{2}+\mu_{2}^{2}+\nu_{1}^{2}+\nu_{2}^{2}$.

For inverted oscillators with Hamiltonian (69), the initial center-of-mass tomogram given by (79) takes the form of conditional probability distribution of one random variable *X*

(82) wcm,α(X|μ1,ν1,μ2,ν2,t)=1πσN±2(α)exp−(X−2Reα1(μ1cosht+ν1sinht)−2Reα2(μ2cosht+ν2sinht)−2Imα1(μ1sinht+ν1cosht)−2Imα2(μ2sinht+ν2cosht))2/σ±exp−2|α1|−2|α2|−(X−i2Imα1(μ1cosht+ν1sinht)−i2Imα2(μ2cosht+ν2sinht)+i2Reα1(μ1sinht+ν1cosht)+i2Reα2(μ2sinht+ν2cosht))2/σ±exp−2|α1|−2|α2|−(X+i2Imα1(μ1cosht+ν1sinht)+i2Imα2(μ2cosht+ν2sinht)−i2Reα1(μ1sinht+ν1cosht)−i2Reα2(μ2sinht+ν2cosht))2/σ+exp−(X+2Reα1(μ1cosht+ν1sinht)+2Reα2(μ2cosht+ν2sinht)+2Imα1(μ1sinht+ν1cosht)+2Imα2(μ2sinht+ν2cosht))2/σ,

where $\sigma =\cosh2t\left(\mu_{1}^{2}+\mu_{2}^{2}+\nu_{1}^{2}+\nu_{2}^{2}\right)+2\sinh2t\left(\mu_{1}\nu_{1}+\mu_{2}\nu_{2}\right)$.

## 9. Conclusions

For almost a hundred years since the discovery and development of quantum mechanics, there has been a problem with understanding its foundations and mysteries. One of the main mysteries is the following problem. Why a classical object, such as the Moon orbiting the Earth, exhibits behavior that we understand. Newton’s law of classical elliptical motion ensures that the Moon rotates around the Globe with constant energy. This is intuitive and fits our daily experience. However, since the beginning of the quantum age, we find that the electron in the hydrogen atom revolves around the proton and has a spectrum of energy levels that is completely contrary to our classical intuition. To understand this quantum behavior, a wave function dynamics formalism was developed that obeys the Schrödinger equation and completely contradicts our classical intuition. The novelty of this article lies in the fact that the results obtained within it open up the possibility of extending them to all other quantum systems, and thus enhancing our understanding of quantum phenomena, closer to the classical intuition. As an alternative to wave functions and density operators describing the states of quantum objects, in the last century, a probability distribution was proposed to describe the states of electrons.

Of course, quantum objects, such as electrons, move around protons according to the equations of quantum evolution, such as the Schrödinger equation. They have energy corresponding to quantized energy levels, and their trajectories do not coincide with the trajectories of the Moon’s rotation around the Globe. In this sense, there is no direct similarity between the quantum dynamics of electrons and the classical dynamics of the Moon. However, the similarity between understanding quantum motion and classical motion lies in the fact that both the motion of the Moon and the motion of electrons can be described by conventional probability distribution functions.

This description is closer to our intuition and experience of the statistical description of random motions. In science, this description has been the subject of lengthy research, and over the past two decades it has been achieved. The time evolution of oscillators, both conventional and inverted, is also studied in the present paper. This consideration opens up the possibility of extending the probabilistic approach describing quantum states to all other systems in order to apply these results to the development of new quantum technologies, such as quantum computing, quantum information technology, etc.

To conclude, we summarize the main results of our paper. We developed the probability representation of quantum states in which the system states are described by standard probability distribution functions. These functions determine the density operators of the states. For this, we considered two different schemes of such construction, namely, symplectic tomography probability distributions [13] and center-of-mass tomographic probability distributions [56]. In our work, we considered time evolution of the tomographic probability distributions, using an example of the Schrödinger cat states of the two-mode oscillator. The main aim was to determine the time evolution and the explicit expressions of center-of-mass tomographic probability distributions for even and odd coherent states of two-mode oscillators in ordinary (81) and inverted (82) forms. The main result is that the obtained probability distributions describe the entangled states of two-mode oscillator and its evolution. In an example of such a state, we constructed the entangled probability distributions and their dynamics. Furthermore, we studied the structure of generic entangled probability distributions. The entangled probability distributions are the new forms of standard probability distributions [12]. The possibility to construct such new probability distributions can be studied considering multi-mode oscillators with time-dependent parameters. The entangled probability distributions are new forms of distributions introduced using quantum mechanics. There are other new aspects of the classical probability theory that can be found and formulated in view of the existence of the quantum formalism of the Hilbert spaces and operators acting in the Hilbert spaces, such as Bell inequalities, which can be considered as consequences of the entangled probability distributions as well as several entropic inequalities that are obvious in quantum mechanics; however, these relations are poorly clarified, and were not even discussed in the classical probability theory. We will consider these problems and entropic properties of such probability distributions in future publications.

## Data Availability

Not applicable.
